# Supplementary material for: Effects of IGF1 rs6214 Polymorphism and Milk Consumption on Serum Levels of IGF-1 and GH and Body Composition
Source: Metabolites. 2025 Aug 20;15(8):556. doi: 10.3390/metabo15080556 (PMC12388142; doi:10.3390/metabo15080556)
Supplement: Supplementary file 1 [file metabolites-15-00556-s001.zip › metabolites-3681399-supplementary.pdf]

DAIRY CONSUMPTION FINDING

INDICATIONS: PLEASE "UNDERLINE" OR "MARK" THE ANSWER THAT BEST DESCRIBES YOUR ANSWER TO EACH QUESTION

1. HAVE YOU CONSUMED DAIRY PRODUCTS IN YOUR LIFE? YES NO
2. DO YOU HAVE ANY LIVER PROBLEMS? YES NO
3. DO YOU CONSUME ALCOHOLIC BEVERAGES? YES NO
4. HOW OFTEN DO YOU CONSUME ALCOHOLIC BEVERAGES?
   a. NULL
   b. 1-3 TIMES PER WEEK
   c. 5-7 TIMES PER WEEK
5. WHEN YOU WERE A CHILD, DID YOU CONSUME DAIRY PRODUCTS? YES NO
6. WHAT DAIRY PRODUCTS DID YOU CONSUME IN YOUR CHILDHOOD?
   a. MILK
   b. CHEESE
   c. BUTTER
   d. YOGURT
7. HOW FREQUENTLY DID YOU CONSUME ANY OF THESE PRODUCTS IN YOUR CHILDHOOD?
   a. NULL
   b. 1-3 TIMES PER WEEK
   c. 5-7 TIMES PER WEEK
8. DO YOU HAVE ANY THYROID-RELATED PROBLEMS? YES NO
9. DID YOU CONSUME MILK IN YOUR CHILDHOOD? YES NO
10. HOW MUCH MILK DID YOU CONSUME IN YOUR CHILDHOOD?
    a. NULL
    b. 1-3 TIMES PER WEEK
    c. 5-7 TIMES PER WEEK
11. DO YOU CURRENTLY CONSUME DAIRY PRODUCTS? YES NO
12. WHAT TYPES OF DAIRY PRODUCTS DO YOU CURRENTLY CONSUME?
    a. MILK
    b. CHEESE
    c. BUTTER
    d. YOGURT
13. HOW OFTEN DO YOU CONSUME THESE TYPES OF PRODUCTS?
    a. NULL
    b. 1-3 TIMES PER WEEK
    c. 5-7 TIMES PER WEEK
14. DO YOU TAKE ANY HORMONAL MEDICATION? YES NO
15. DO YOU ENGAGE IN ANY TYPE OF EXERCISE? YES NO
16. HOW FREQUENTLY DO YOU EXERCISE?
    a. NULL
    b. 1-3 TIMES PER WEEK
    c. 5-7 TIMES PER WEEK
17. DO YOU TAKE ANY SPORTS SUPPLEMENTS? YES NO

18. DO YOU SMOKE? YES NO
19. DO YOU CURRENTLY CONSUME MILK? YES NO
20. HOW MUCH MILK DO YOU CONSUME?
    a. NULL
    b. 1-3 TIMES PER WEEK
    c. 5-7 TIMES PER WEEK
21. WHAT TYPE OF MILK DO YOU CONSUME?
    a. WHOLE
    b. LACTOSE-FREE
    c. LIGHT
    d. SKIMMED
    e. SEMI-SKIMMED
